# Comparative chloroplast genomics of the genus *Taxodium*

**DOI:** 10.1186/s12864-020-6532-1

**Published:** 2020-01-31

**Authors:** Hao Duan, Jinbo Guo, Lei Xuan, Ziyang Wang, Mingzhi Li, Yunlong Yin, Ying Yang

**Affiliations:** 1 Jiangsu Engineering Research Center for Taxodium Rich, Germplasm Innovation and Propagation, Institute of Botany, Jiangsu Province and Chinese Academy of Sciences (Nanjing Botanical Garden Mem, Sun Yat-Sen), Nanjing, China; 2 Biodata Biotechnology Co. Ltd, Hefei, China

**Keywords:** *Taxodium*, chloroplast genome, repeat, indel, single nucleotide polymorphisms, arrangement

## Abstract

**Background:**

Chloroplast (cp) genome information would facilitate the development and utilization of *Taxodium* resources. However, cp genome characteristics of *Taxodium* were poorly understood.

**Results:**

We determined the complete cp genome sequences of *T. distichum*, *T. mucronatum*, and *T. ascendens*. The cp genomes are 131,947 bp to 132,613 bp in length, encode 120 genes with the same order, and lack typical inverted repeat (IR) regions. The longest small IR, a 282 bp trnQ-containing IR, were involved in the formation of isomers. Comparative analysis of the 3 cp genomes showed that 91.57% of the indels resulted in the periodic variation of tandem repeat (TR) motifs and 72.46% single nucleotide polymorphisms (SNPs) located closely to TRs, suggesting a relationship between TRs and mutational dynamics. Eleven hypervariable regions were identified as candidates for DNA barcode development. *Hypothetical cp open reading frame 1*(*Ycf1*) was the only one gene that has an indel in coding DNA sequence, and the indel is composed of a long TR. When extended to cupressophytes, *ycf1* genes have undergone a universal insertion of TRs accompanied by extreme length expansion. Meanwhile, *ycf1* also located in rearrangement endpoints of cupressophyte cp genomes. All these characteristics highlight the important role of repeats in the evolution of cp genomes.

**Conclusions:**

This study added new evidence for the role of repeats in the dynamics mechanism of cp genome mutation and rearrangement. Moreover, the information of TRs and hypervariable regions would provide reliable molecular resources for future research focusing on the infrageneric taxa identification, phylogenetic resolution, population structure and biodiversity for the genus *Taxodium* and Cupressophytes.

## Background

*Taxodium* belongs to the family Cupressaceae, is native to North America and Mexico, and contains three tax: bald cypress, pond cypress and montezuma cypress. However, there have been continuous debates concerning the taxonomy of these three taxa as one, two, or three species from the nineteenth century to the present [1–3]. In this study, we temporarily consider treating them as three species. *Taxodium* have strong resistance to biotic and abiotic stresses, and their life span can be as long as thousands of years [4]. Since 1973, Institute of Botany, Jiangsu Province and Chinese Academy of Sciences has been vigorously engaged in interspecific hybridization breeding of *Taxodium*. A batch of new varieties named ‘Zhongshanshan’ have been selected from the hybrids with the advantages of high ornamental value, rapid growth and strong stress resistance. They have been popularized and applied in 18 provinces and municipalities of China, bringing better ecological and social benefits in the urban landscaping, ecological civilization construction, sponge city construction, and ecological restoration of the Yangtze River economic zone [4]. ‘Zhongshanshan’ has become an important tree species with huge market demand in China. Although it has great economic value for the development and utilization of *Taxodium* resources, the research basis of phylogenetics, species/variety identification and genetic diversity of this genus is weak at present.

As an semi-autonomous replication organelles, the chloroplast genome has some unique advantages compared with nuclear and mitochondrion genome [5]. Cp genome is much smaller than the nuclear genome, and it’s easy to obtain the complete cp genome sequence. The gene density of cp genome is larger and the evolution rate is moderate, and segments with different evolution rates can be selected for different research purposes. The cp genomes of higher plants are highly conserved in organization, gene order and content, which can ensure homology among distant evolutionary groups. Besides, genes of cp genomes are single-copy, which ensures the direct homology of genes among species, and there is almost no interference of side-line homologous genes. Therefore, cp genome has unique value in phylogenetics, species identification and population genetics of higher plants.

Typically, the circular genomes of cp are organized into large and small single-copy regions, separated by an inverted repeat (IR). IR region is a pair of sequences with the same sequence and opposite direction, named IR_A_ and IR_B_ respectively. The sequence between IR_A_ and IR_B_ can produce triggered flip-flop recombination, which can stabilize the single-copy regions. Although the gene content and arrangement of cp genomes are relatively conservative, a series of changes have taken place from point to surface, including RNA editing (base insertion/deletion and substitution/transition), gene transfer and loss, inversion events, etc. The typical feature of the cp genomes of conifers were the loss of IR region [6]. Some small inverted repeats (sIRs) were found in cp genomes facilitating the stabilization. Wu et al. analyzed the plastomes of 24 representative genera in all of the five cupressophyte families, and found that every cupressophyte family has evolved its own specific and novel sIR systems, for example the rpoC2-IR in *Sciadopitysis* [7], the trnN-IR in *Podocarpaceae* [8], and the rrn5-IR in *Araucariaceae* [8]. TrnQ-UUG containing sIRs were found in almost all families of Cupressaceae and Taxaceae, except Callitris [8], and were found mediating HR [6, 9, 10]. Some other sIRs can also mediate HR in *Cupressaceae* and *Taxaceae*. For example, a 335 bp *trnN*-GUU containing sIR and a 211 bp sIR in the IGS of *Torreya fargesii* [10]. Due to the loss of IR region, conifer cps are also characterized by the extensive genomic rearrangements compared with most angiosperms [11]. The mechanisms underlying indel (insertion/deletion), Single nucleotide polymorphism (SNP) and rearrangement of cp genomes have attracted the attentions of many researchers [12–14].

Wu et al. [8] published the cp genome of *Taxodium distichum*, which is the only published cp genome of *Taxodium*. Nevertheless, the aim of its development is to systematically study the high variation in cp size and organization of Conifers II (cupressophytes) [8]. Changes in the gene and structure of cp genomes in the genus has not been referred. Due to the controversial taxonomy of the genus *Taxodium*, it is impossible to determine which taxa the published chloroplast genome belongs to. Therefore, in addition to development of cpDNA of *T. ascendens* and *T. mucronatum*, the cp genome of *T. distichum* was also re-sequenced in this study. We analyzed the structure characteristics of *Taxodium* cp genomes, conducted comparative analysis between the 3 cp genomes, and look insights into the dynamics of cp genome mutation and rearrangement of *Taxodium*. The results would advance our current understanding of the complexity, dynamics, and evolution of cp in conifers.

## Results

### Sequencing of *Taxodium* plastid genomes

**Fig. 1:**
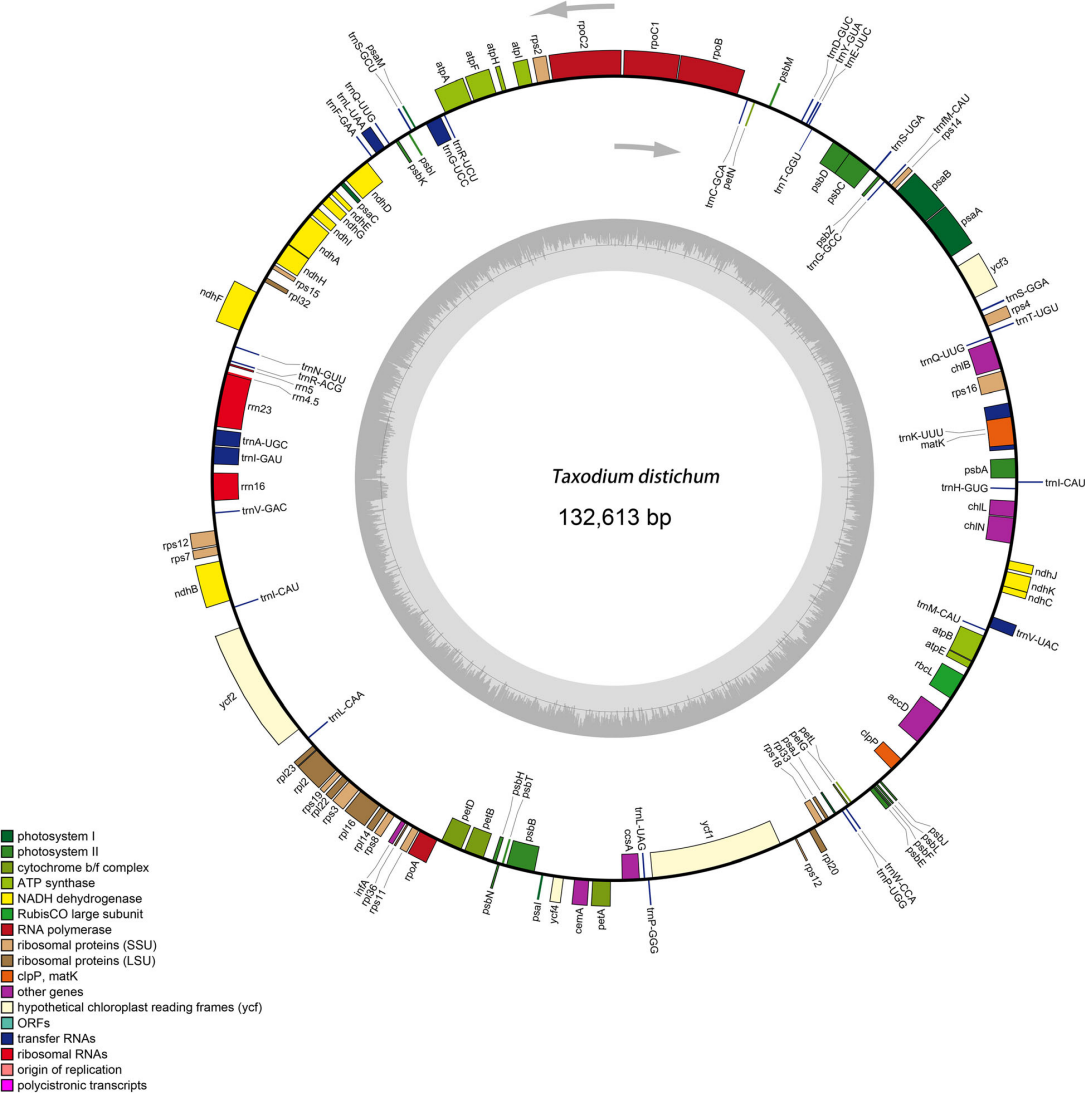
Circular gene map of the chloroplast genome of *T. ascendens.* Genes drawn within the circle are transcribed clockwise, while those drawn outside are transcribed counterclockwise. Genes are color-coded according to their functional groups. Inner circle represents GC content

Illumina 150-bp paired-end sequencing of long-range PCR-amplified plastid DNA generated 5045–6946 Mb clean reads for the three sampled *Taxodium* species (Table 1). Using the combination of de novo and reference-guided assembly, we obtained complete plastid nucleotide sequences for all three species. The nucleotide sequences of the four plastid genomes range from 131,947 bp in *T. distichum* to 132,613 bp in *T. ascendens* (Table 2). Like the cp genomes of other cupressophyte species, they lack the IR region and have no distinct quadruple structure. The gene map of the *T. ascendens* plastid genome is presented in Fig. 1 as a representative. The three genomes encode an identical set of 120 genes (Additional file 1), and the arrangements of these 120 genes are totally collinear (Additional file 2). The 120 unique genes include 83 protein-coding genes (Table 2), 33 transfer RNA (tRNA) genes, and 4 ribosomal RNA (rRNA)genes. They also have similar GC contents of 35.22–35.26%. This is similar to other gymnosperm plastid genomes.

**Table 1:** Sequencing and assembly results of three chloroplast genomes of *Taxodium*

Species	Raw data (Mb)	Clean data (Mb)	Clean data GC(%)	Clean data Q20(%)	Clean data Q30(%)	GC Content(%)	N rate (%)
*T. ascendens*	5329	4895	37.85	98.02	93.94	35.22%	0%
*T. distichum*	5045	4695	35.74	98.11	94.2	35.26%	0%
*T. mucronatum*	6946	6595	34.19	98.38	94.91	35.25%	0%

Note: Read length: read length of valid data; Clean data GC: average GC content of valid data; Clean data Q20: Q20 value of valid data; Clean data Q30: Q30 value of valid data. Total Length (bp): The total length of the sample assembly result; GC Content (%): GC content of the sample assembly sequence; N rate (%): the content of unknown base N in the sample assembly sequence

**Table 2:** Gene information statistics

Species	Accession No.	Genome size (bp)	Coding Gene number (#)	CDS total length (bp)	CDS average length (bp)	CDS length / Genome (%)
*T. ascendens*	MN535012	132,613	83	74,469	897	56.16
*T. distichum*	MN535013	131,947	83	74,217	894	56.25
*T. mucronatum*	MN535011	132,037	83	74,217	894	56.21

Accession No.: Accession number of the complete chloroplast genome in genebank database. CDS: coding sequence

### Tandem repeats analysis

**Fig. 2:**
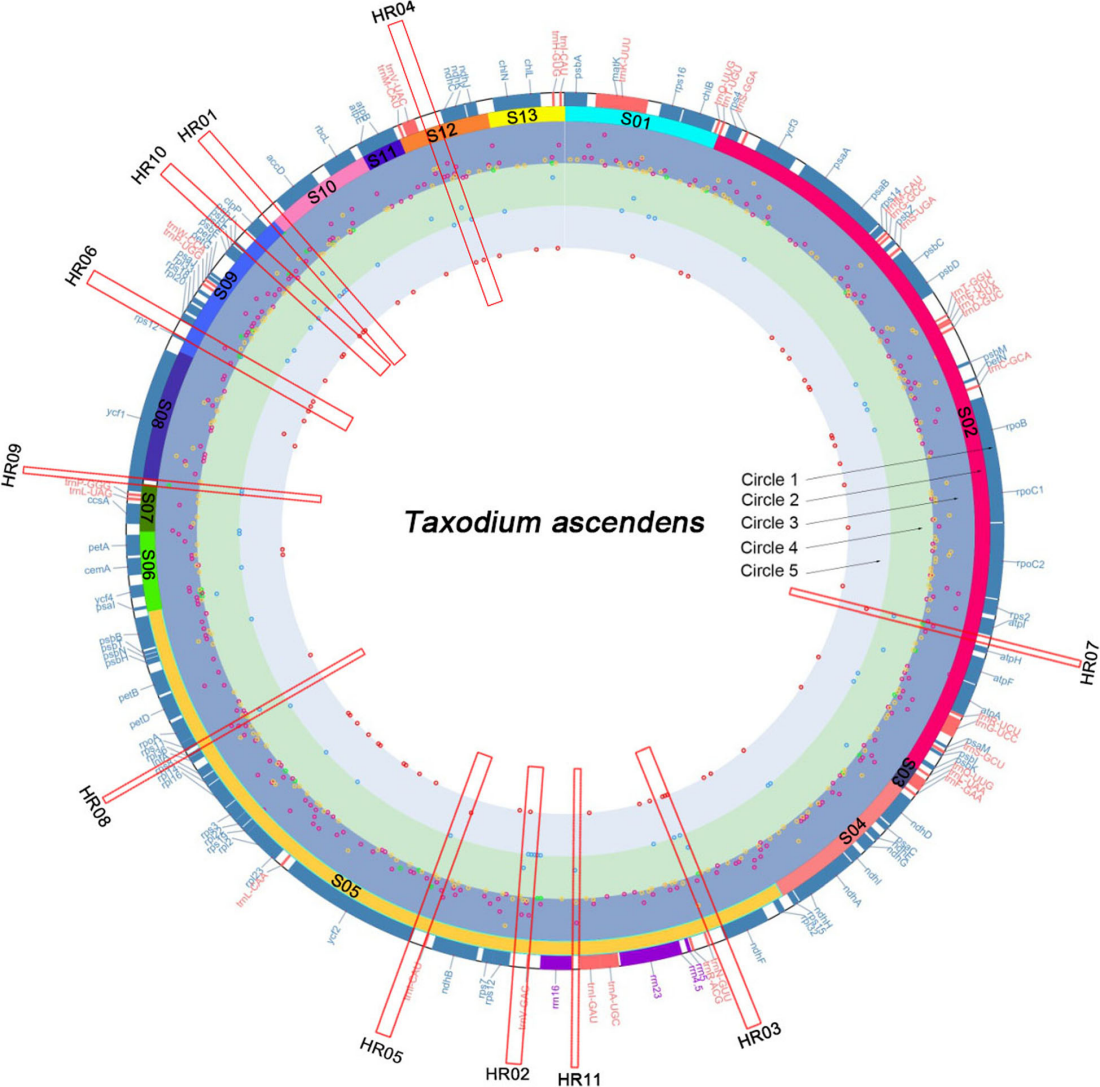
Distribution of conserved gene blocks, TRs, indels, and SNPs in the plastomes of *T. ascendens*. **Circle 1**: plastome map of *T. ascendens* with coding genes labeled in blue, tRNAs labeled in red, and rRNAs in purple. **Circle 2**: conserved blocks of genes relative to the cp genome of *Cycas*. **Circle 3**: location of 639 TRs reported by phobos software. TRs of different length are marked with different colors, with green representing repeats ≥20 bp, rose red representing 10~19 bp, and orange representing < 10 bp. The relative height of the dot position in the figure represents the relative number of polymorphic loci within non-overlapping bins of 200 bp. Repeats in the three different colors were treated separately in statistical analysis. Dots with a high relative position represent more loci belonging to TRs within the 200 bp window. **Circle 4**: counts of indels (blue). **Circle 5**: counts of SNPs (red). Dots with a high relative position, represent more polymorphic loci in the 200 bp window. The red rectangle (HR01-HR11) showed the locations of the 11 selected hypervariable regions. The number or relative position of dots of indel and/or SNP inner rectangles were higher that outside

A TR is a repetitive sequence of adjacent specific nucleic acid sequence patterns repeated twice or more, including simple sequence repeats (SSR), whose repeat motif is 1–6 nucleotides, and long sequence repeats, whose repeat motif is ≥7 nucleotides. A total of 639 TRs were detected in the *T. ascendens* cp genome using Phobos (Additional file 3); the total length of repeats was 8462 bp, and TRs were widely distributed in the coding and non-coding regions of the cp genome (Fig. 2, Circle 3). Repeated motifs ranged from mononucleotide to 95-nucleotide. Among these, 601 were SSRs and 38 were long sequence repeats. Mononucleotide repeats were the most abundant SSRs, accounting for 38.03% (243) of the total, of which 238 repeat units were A/T and only five were G/C (Additional file 4). Of the 55 (8.61%) dinucleotide repeats, 34 were AT/TA type, 17 were AG/TC type, four were AC/TG type, and none were GC/CG type. For trinucleotide repeats, except for four AGC/TCG and two AGG/TCC types, the rest were all repeat types with A/T ratio higher than GC. Among the 79 (12.36%) tetranucleotide repeats, 64 had a higher A/T ratio than GC. Because of the high A/T content of repeat motifs, an increase in repeats will lead to a low GC content of chloroplast genes. The total length of repeats ranged from 7 bp to 453 bp, of which 325 (50.86%) were short and less than 10 bp, 286 (44.76%) were medium-sized and TRs ranging from 10 bp to 20 bp, and 28 (4.38%) were long and TRs ranging from 20 bp to 50 bp. Long TRs were mostly distributed in non-coding regions (Fig. 2, Circle 3, Green dots). The total length of seven long TRs was more than 50 bp (Table 3). Their total lengths were 98 bp (ycf2), 110 bp (psbJ-clpP), 116 bp (rps18), 145 bp (trnI-ycf2), 152 bp (clpP-accD), 333 bp (ycf1) and 453 bp (clpP-accD) (Additional file 3).

**Table 3:** Basic information for long tandem repeats > 50 bp

Repeat Class	Minimum	Maximum	Length	Location	Normalised Repeat Length	Percentage Perfection
95-nucleotide Repeat	116,478	116,930	453	clpP-accD	453	99.78%
63-nucleotide Repeat	101,809	102,141	333	ycf1	333	100%
38-nucleotide Repeat	115,180	115,289	110	psbJ-clpP	110	98.18%
32-nucleotide Repeat	75,866	75,963	98	ycf2	96	94.79%
24-nucleotide Repeat	110,842	110,957	116	rps18	116	96.55%
22-nucleotide Repeat	73,789	73,933	145	trnI-ycf2	145	98.62%
19-nucleotide Repeat	116,921	117,072	152	clpP-accD	152	100%

**Fig. 3:**
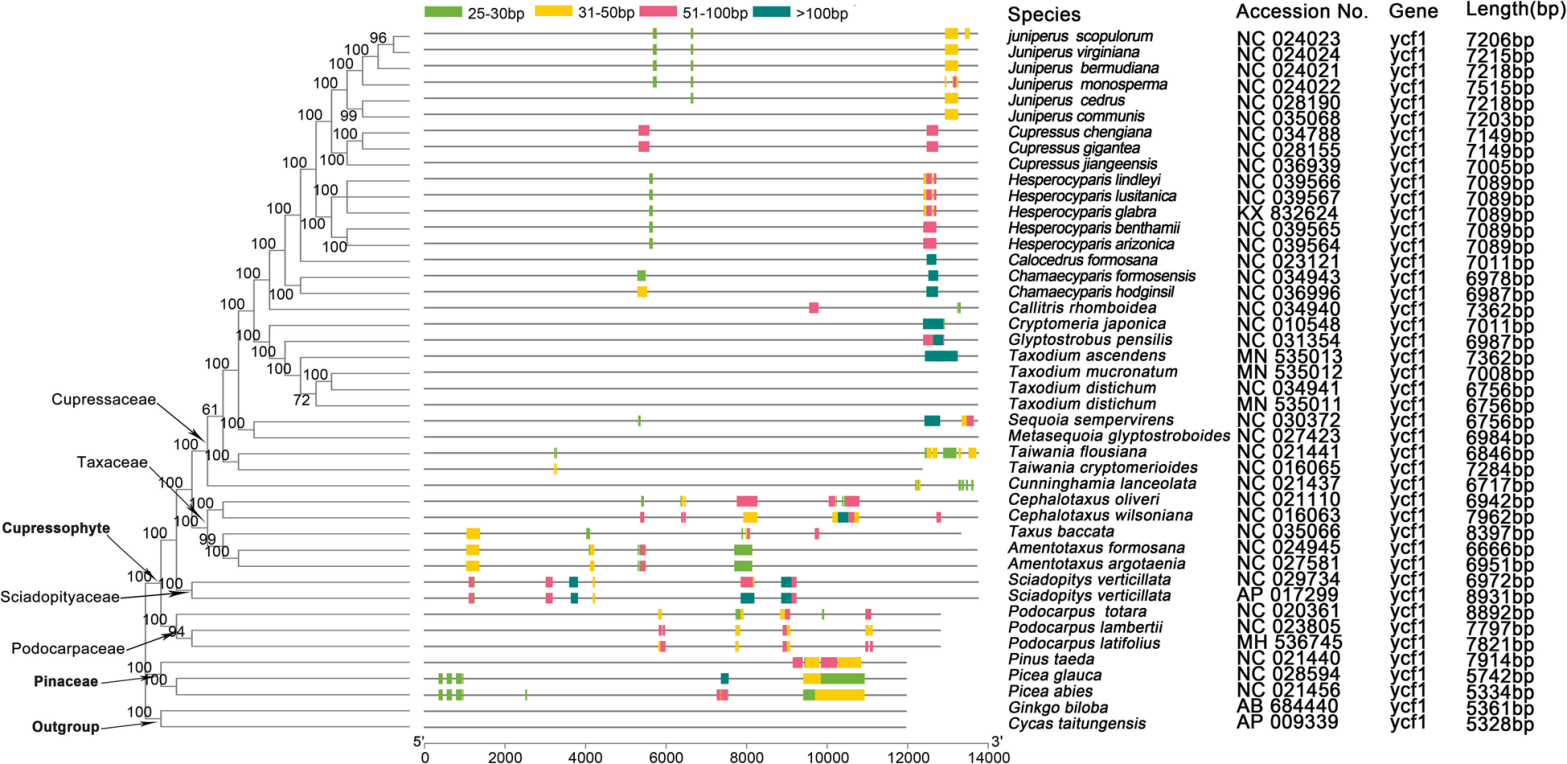
The tandem repeats of ycf1 gene in conifers. The phylogenetic tree was constructed based on the sequence alignments of ycf1 genes using Mafft. The right side of the phylogenetic tree showed the position of tandem repeats on the ycf1 gene. The length of the horizontal line was drawn according to the length of multiple sequence alignment, which included the length of gaps. Therefore, the position of repeats in different species can be mapped to each other. The rightmost column listed the actual length of *ycf1* genes (excluding gaps)

Researches have shown that there are many TRs on the coding DNA sequence (CDS) of *accD* gene and its surrounding regions of gymnosperms [12]. In order to study the general features of *hypothetical cp open reading frame 1*(*ycf1*) genes in cupressophytes, we analyzed the *ycf1* gene sequences in 44 species (Fig. 3). The length of *ycf1* gene in Pinaceae is similar to that of *Ginkgo biloba* and *Cycas*, which were about 5000 bp. However, the *ycf1* gene length in cupressophytes experienced an extraordinary expansion, ranging from 6666 bp to 8931 bp, with *Taxus baccata* and *Sciadopitys verticillata* has the shortest and longest CDS, respectivelly. There were no TRs in *Ginkgo biloba* and *Cycas* cp gemones, but, except for four species, TRs were detected in most conifers. In the *Taxodium*, TRs were only detected in *T. ascenden.* The same situation happened in *Cupressus*, with TRs are detected in *Cu. chengiana* and *Cu. gigantea* but not in *Cu. jiangeensis*. It can be seen from Fig. 3 that the insertion positions of TRs on the *ycf1* CDS were family specific. For *Cupressaceae*, there are two major insertion positions, one located in the middle of the CDS, the other one is near the C-terminal region.

### Dispersed repeats

Fifty dispersed repeats were detected in the *T. distichum*, *T. mucronatum*, and *T. ascenden* cp genomes, respectively, using REPuter (Additional file 5). In the *T. distichum* cp genome, there were 24 forward repeats, 21 palindromic repeats, three complement repeats, and two reverse repeats. In the *T. mucronatum* cp genome, there were 26 forward repeats, 18 palindromic repeats, one complement repeat, and five reverse repeats. In the *T. ascendens* cp genome, only 36 forward repeats and 14 palindromic repeats were detected.

In cupressophyte cp genomes, the highly reduced IRs are replaced by short repeats that have the potential to mediate homologous recombination [6, 9, 10]. Three sIRs >100 bp were detected in *T. ascendens* (Table 4), and their sequences were identical in all three *Taxodium* cp genomes. Among them, sIR1 and sIR3 contained complete trnQ-UUG and trnI-CAU genes, respectively. For sIR2, one copy was located in the intergenic region (IGS) region of *petA*-*ccsA* and the other in the *psbJ*-*clpP* IGS.

**Table 4:** SIRs in the cpDNA of *Taxodium ascendens*

Name	Copy	Location	Length	Mismatch	Contained gene
sIR1	a	7412–7693	282	3	trnQ-UUG
	b	45,569–45,850			trnQ-UUG
sIR2	a	99,641–99,759	119	2	Null (PetA-ccsA)
	b	115,292–115,410			Null (psbJ-clpP)
sIR3	a	73,129–73,241	111	3	trnI-CAU
	b	132,385–132,497			trnI-CAU

**Fig. 4:**
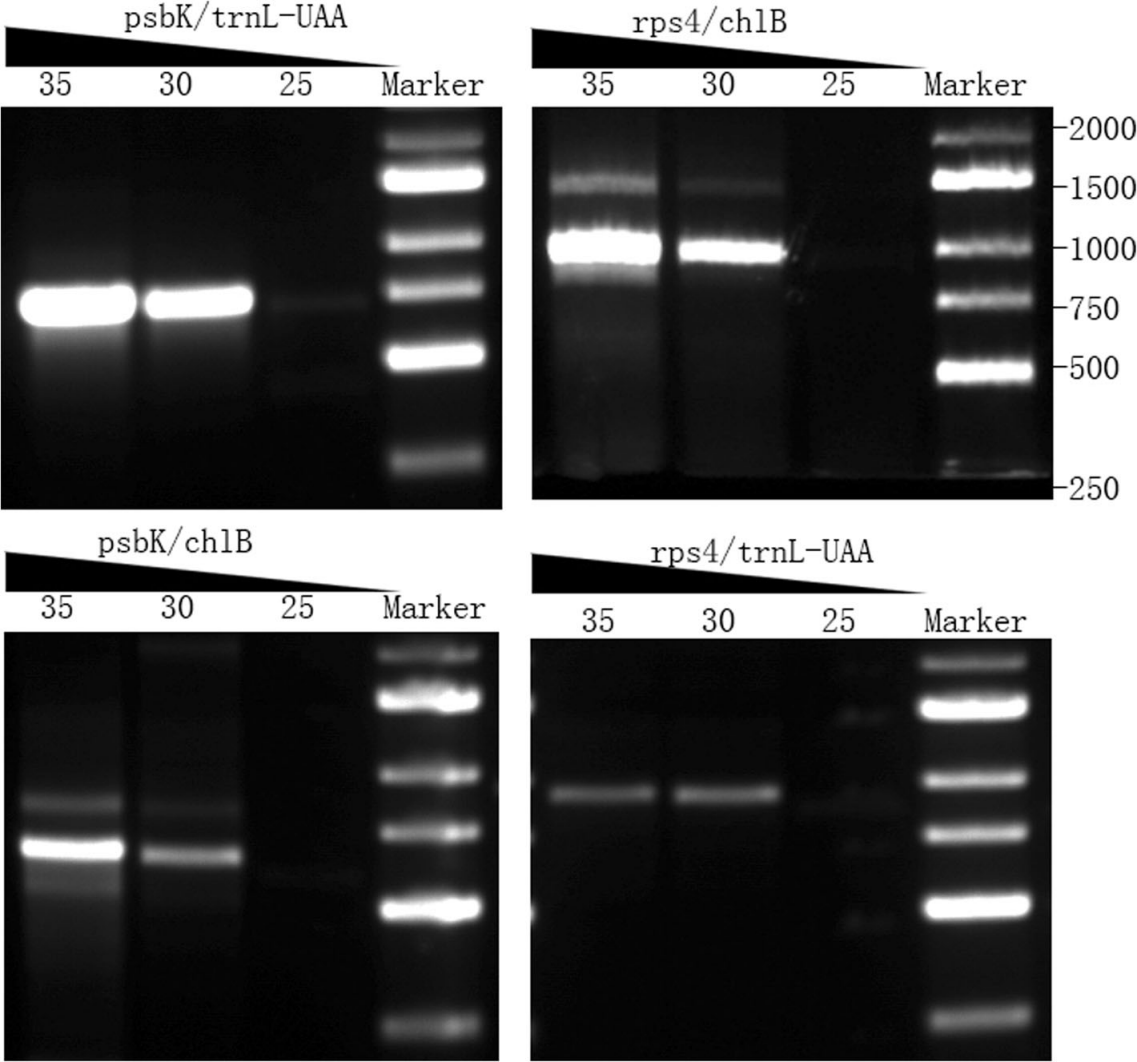
Co-existence of two isomeric chloroplasts in *T. distichum*. The corresponding PCR amplicons are shown, and the numbers above each lane of gel photos denote the PCR cycles conducted

The *trnQ*-UUG gene was the only one in a sIR longer than 200 bp. If the 282-bp IR is able to mediate homologous recombination (HR), we would expect the presence of two type isomers. Semi-quantitative PCR with a variable number of cycles was conducted to verify the presence of the two isomers. The isomers illustrated in Fig. 1 is designated as the type I, and the other is the type II. All four reactions generated products, which verified the presence of both the I and II forms. It was also apparent that there were minor differences in amplification efficiency between the four PCR reactions. With 30 PCR cycles, the electrophoresis bands of type 1(*rps4*/*chlB* products) are very bright, while those of type 2(*psbK*/*trnL* products) are much weaker (Fig. 4). These results suggest that the type I is predominant in *T. ascendens*, in agreement with our assembly results.

To quantify the relative frequency of the two isomeric genomic forms, Illumina paired-end reads were mapped to the genome and isomer frequencies were calculated using the method of Guo [9]. There were 297 read pairs that spanned the trnQ-containing IR copies, of which 293 pairs (98.65%) supported the type I isomer while four pairs (1.35%) supported the type II isomer.

### Phylogenetic and rearrangements analysis

**Fig. 5:**
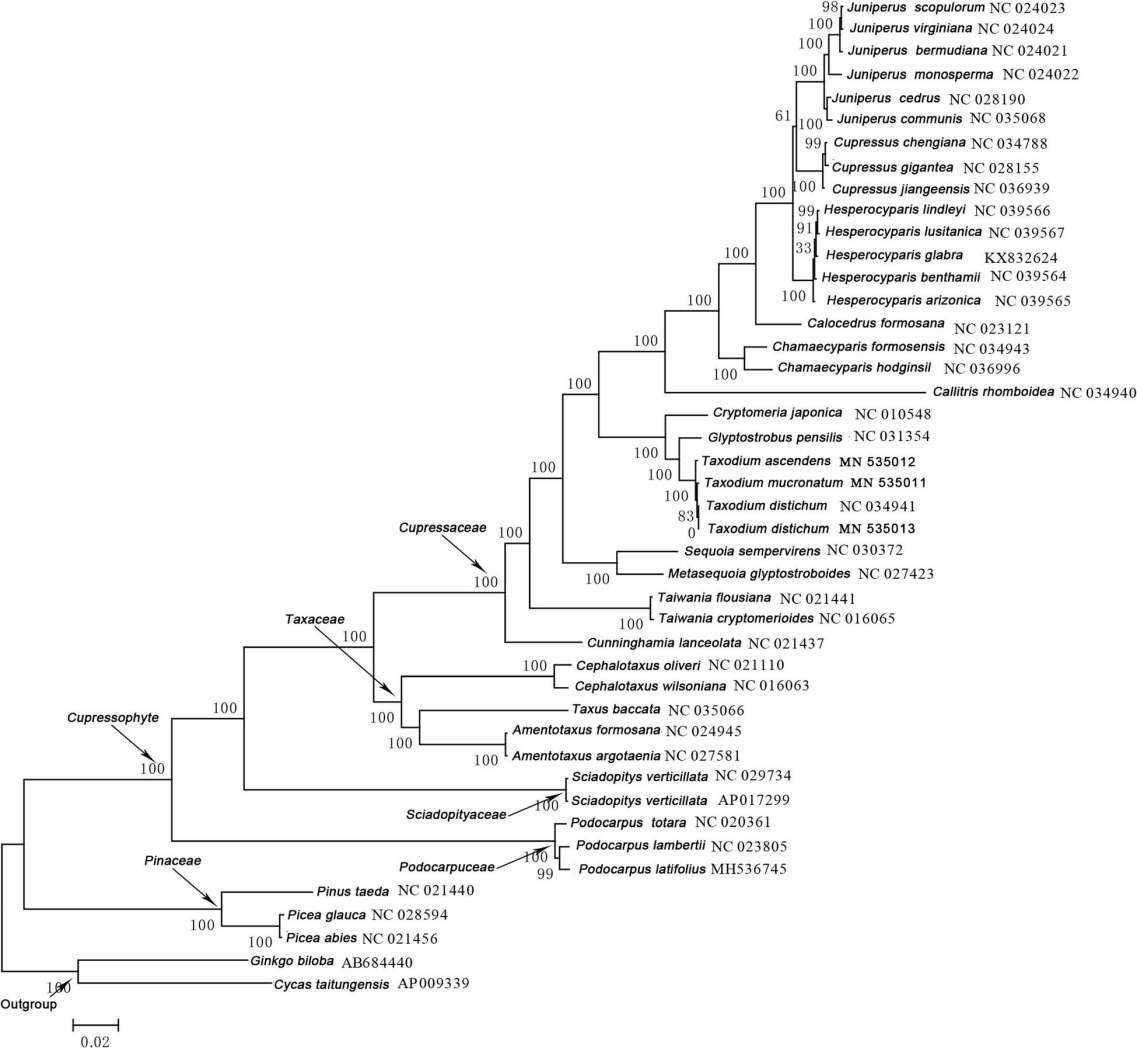
Phylogenetic analysis of 44 chloroplast genomes. Numbers below each node are bootstrap support values

Phylogenetic tree based on the 51 single copy coding genes of 44 species were constructed (Fig. 5). Among the genus *Taxodium*, *T. mucronatum* and *T. distichum* were clustered together. The genus *Taxodium* has the closest relationship with *Glyptostrobus*, and then forms a group with *Cryptomeria*. Unanimously, mauve alignment showed that no rearrangements occurred between *Taxodium* and *Glyptostrobus pensilis*, while at least four rearrangements occurred between *Taxodium* and *Cryptomeria japonica* (Additional file 6)*.*

**Fig. 6:**
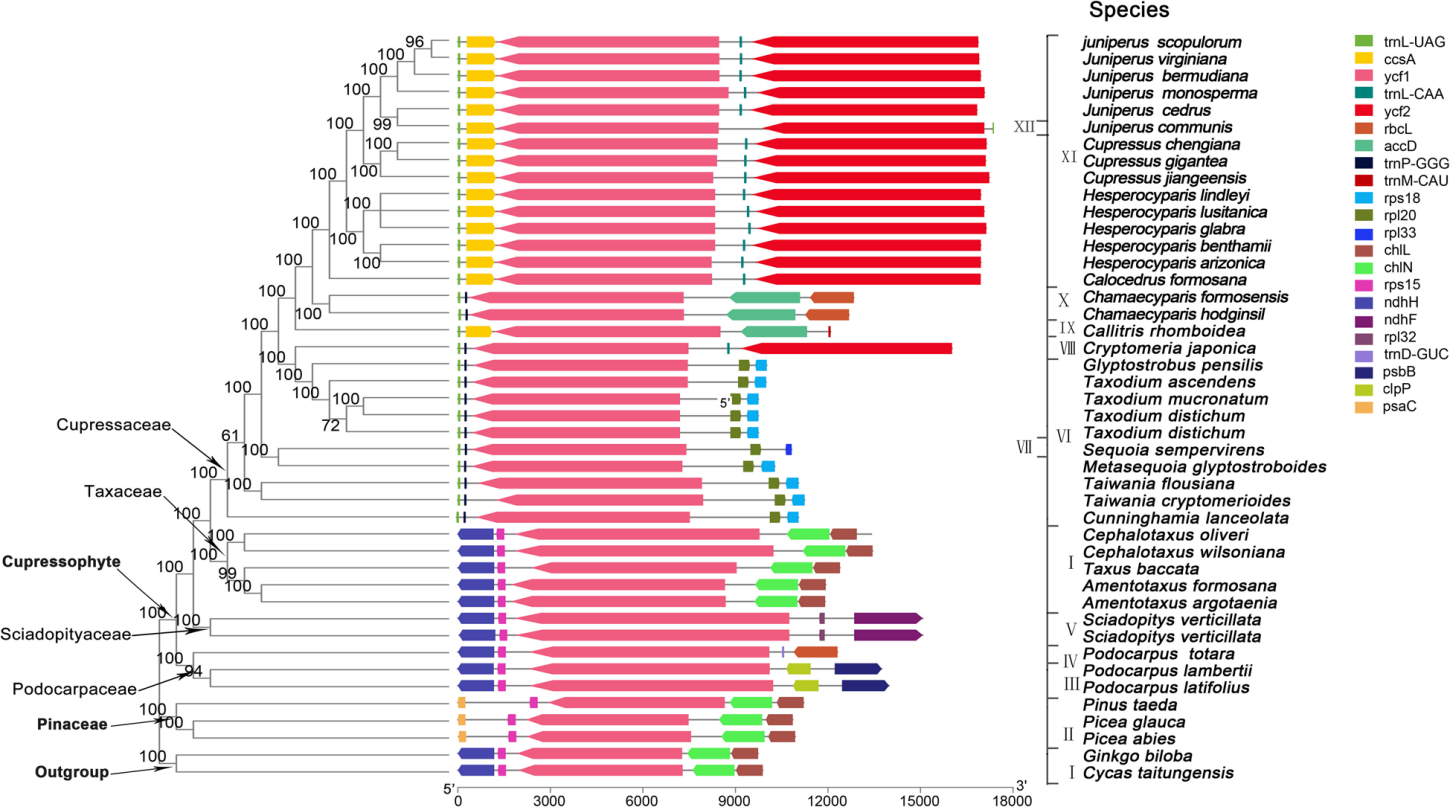
Gene organization around the *ycf1* gene in gymnosperms. The direction of the arrow of genes was from the N-terminal to the C-terminal. The roman numbers I–XII denotes 12 types of gene organization around the *ycf1*

Previous studies have shown that cycads possess the oldest sequence of genes in seed plants. We conducted mauve alignment between *Taxodium* and *Cycad taitungensis* cp genomes. Compared with *Cycas*, there were 13 conserved gene clusters in *Taxodium* cp genomes, which were labeled S01 to S13 (Table 5) (Fig. 2, Circle 2). Therefore, there were at least 13 rearrangements in the process of transformation from cycad chloroplast genome structure to *T. ascendens* genome structure. The size of the conversed gene blocks ranged from 1236 bp to 40489 bp. Five of the 13 inversion endpoints occurred near tRNAs, including *trnI*, *trnT*, *trnQ*, *trnF*, and *trnM*. Interesting, there is a sequence between S07 and S08 that can't find homologous sequence on *Cycad*, and its position (101807-102061) overlaped with the 63-nucleotide repeat TR493 (101809-102141) on ycf1(Additional file 3). Two other inversion endpoints were also in TRs. The inversion endpoint (99473) between conversed gene blocks S07 and S08 was in the mononucleotide repeat TR484 (99466-99478) located in *petA*-*ccsA*. The inversion endpoint (116761) between conserved gene blocks S10 and S11 was in the 95-nucleotide repeat TP571 (116478-116930) located on *clpP*-*accD*.The *accD* gene or its adjacent region is a hot rearrangement area of cupressophyte cp genomes, Li et al. found that there are five types of gene order in cupressophytes, and speculated that many inversion events have occurred here during the evolution of cupressophyte cp genomes [12]. We also analysis the sequence variability of the genes adjacent to the *ycf1* gene in *Taxodium*. The gene order around *ycf1* of the 44 analyzed species could be classified into twelve types (Fig. 6). Cycas, Ginkgo, and Taxaceae have the same gene order: ndhH-rps15-ycf1-chlN-chlL gene order (type I). As we can see from Fig. 6, at least one side (right side/left side) of *ycf1* gene in type II (Pinaceae), type III (Podocarpuceae), type IV (Podocarpuceae) and type V (Podocarpuceae) maintains the same gene order of type I. However, in Cupressaceae (type VI to XII), gene orders of both sides (right side and left side) of *ycf1* gene were totally different from type I. Therefore, the arrangement frequency around *ycf1* gene in Cupressaceae was much higher. In the Cupressaceae family, *Glyptostrobus*, *Taxodium*,*Metasequoia*, *Taiwannia* and *Cunninghamia* have a conversed trnL (UAG)- trnP (GGG)-ycf1-rpl20 –rps18(type VI) gene order. And in *Juniperus*, *Cupressus* and *Hesperocyparis*, the gene order is mainly: trnL (UAG)-ccsA-ycf1-trnL (CAA)-ycf2(type XI). In view of the diversity of gene organization around *ycf1* gene, it is speculated that *ycf1* gene may be frequently involved in the rearrangement events of *cupressophytes cp ge*nomes.

**Table 5:** Information of the 13 conserved gene blocks of *Taxodium* cp genomes compared with *Cycas taitungensis*

Name	Start	End	Length (bp)
S01	1(trnI-psbA)	7831(trnT)	7831
S02	7832(trnT)	45,722(psbK-trnQ)	37,891
S03	45,723(psbK-trnQ)	46,958(trnF-ndhD)	1236
S04	46,959(trnF-ndhD)	54,967(rps15-rpl32)	8009
S05	54,968(rps15-rpl32)	95,456(psbB-psaI)	40,489
S06	95,457(psbB-psaI)	99,472(petA-ccsA)	4016
S07	99,473(petA-ccsA)	101,806(ycf1)	2334
S08	102,062(ycf1)	108,715(ycf1-rpl20)	6654
S09	108,715(ycf1-rpl20)	116,760(clpP-accD)	8045
S10	116,761(clpP-accD)	122,206(rbcL-atpE)	5446
S11	122,207(rbcL-atpE)	124,233(atpB-trnM)	2027
S12	124,234(atpB-trnM)	128,797(ndhJ-chlN)	4564
S13	128,798(ndhJ-chlN)	132,613(trnI- psbA)	3815

#### Comparative analysis of genomic structure

Compared with *T. ascendens*, 83 indels, including 43 deletions and 40 insertions of different origins were detected in *T. distichum* and *T. mucronatum* (Fig. 2, Circle 4). Among them, 82 indels occurred in IGS regions, and only one 252 bp indel occurred in the CDS region of ycf1. Therefore, the total CDS length of *T. ascendens* is 252 bp longer than of *T. distichum* and *T. mucronatum*. The indel did not caused frame shifts or stop codons. Among the 83 indels, only seven (8.43%) were located outside repeat regions, and the remaining 76 (91.57%) were located within 51 TRs (Additional file 7). Among these, 64 indel sequences were integer multiples of repeat motifs, that is, the generation of indel sequences created differences in the number of complete repeat motifs. Twelve indel sequences were non-integer multiples of repeat motifs, i.e., the indel sequences contained partial incomplete repeat motif sequences. Of the 51 TRs containing indels, 47 (92.16%) were SSR indels of 1–4 nucleotides. Among these, 30 belonged to mononucleotide repeat type A/T, 14 belonged to dinucleotide repeat type AT/TA, two belonged to trinucleotide repeat type AAT/ATT, and one belonged to tetranucleotide repeat type AAAT/ATTT. All SSR indels contained only A/T. The remaining four large indels were 19-, 22-, 28-, and 95-nucleotide repeats.

**Fig. 7:**
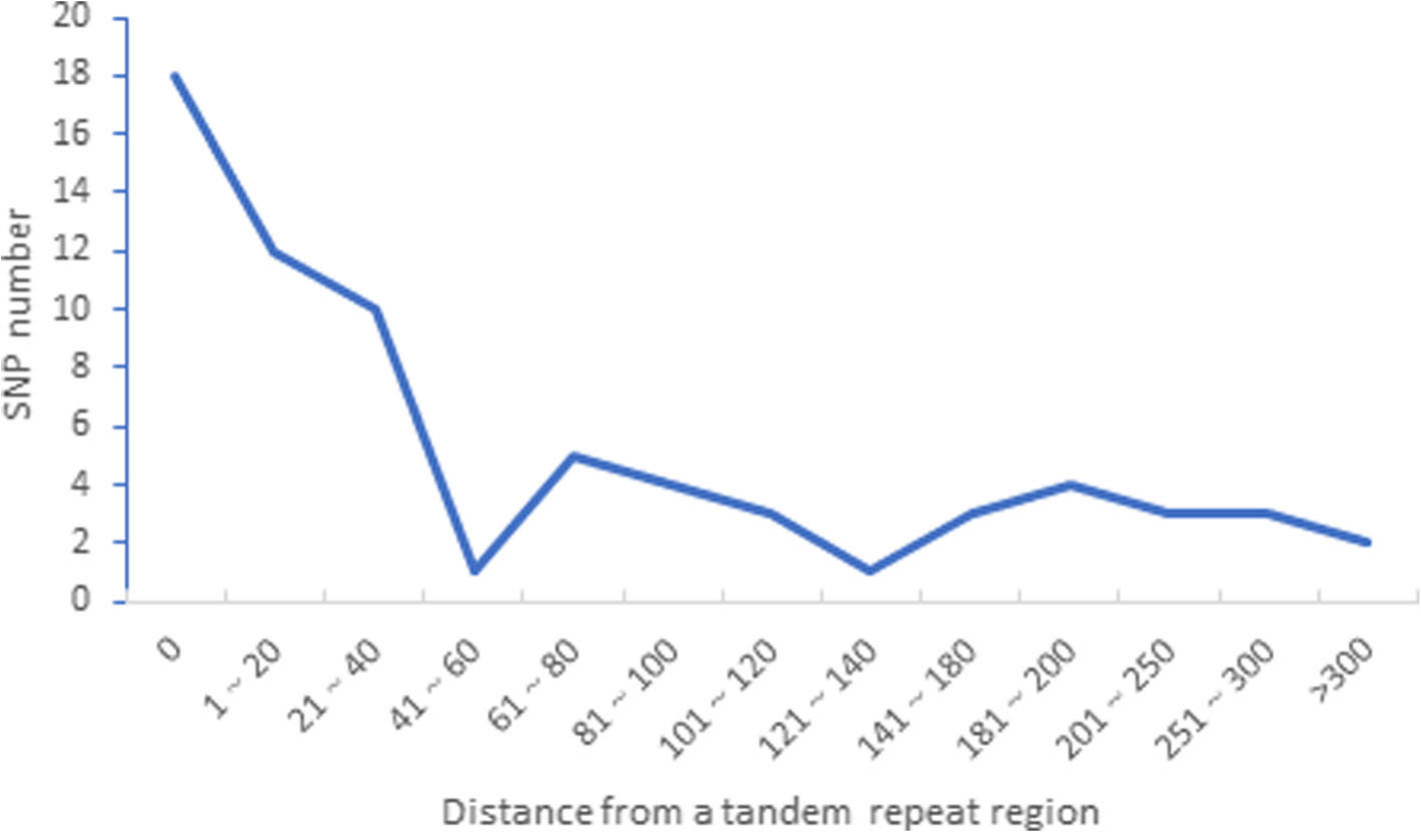
Number of SNPs within 0–300 bp windows adjacent to the tandem repeat regions. The SNPs within 0 bp windows represent SNPs located inside the repeat regions

A total of 45 SNPs were detected in *T. distichum* compared with *T. ascendens*, representing 31 in IGS regions and 14 in CDS regions (Fig. 2, Circle 5), with six synonymous mutations and eight non-synonymous mutations (Table 6). A total of 50 SNPs were detected in *T. mucronatum* compared with *T. ascendens*, representing 35 in IGS regions and 15 in CDS regions, with six synonymous mutations and nine non-synonymous mutations. No mutations appeared on start/stop codon or caused the triplet codon of the site mutates into a termination codon. We merged SNPs occurring at the same site into one for statistical analysis, so a total of 69 SNPs of diverse origin were found. Among these, 45 (65.22%) SNPs were found in non-coding regions and 24 (34.78%) SNPs were found in 16 coding regions, including *rps16*, *psbC*, *rpoB*, *rpoC2*, *ndhA*, *rps12*, *rpl2*, *rps3*, *cemA*, *ycf1*, *clpP*, *accD*, *rbcL*, *atpB*, *ndhK*, and *chlN*. Of the 69 SNPs, 18 (26.09%) were located within the TR sequences, 32 (46.38%) were located within 100 bp windows adjacent to the repeats, and only 19 (27.54%) were located outside the 100 bp windows adjacent to repeats (Fig. 7).

**Table 6:** Statistics of SNP annotation results

	CDS			Intergenic	Total
	Synonymous	Nonsynonymous	Total		
T. distichum	6	8	14	31	45
T. mucronatum	6	9	15	35	50

Note: Synonymous: Synonymous mutation in the gene region; Nonsynonymous: Nonsynonymous mutation in the gene region; Intergenic: SNP in the intergenic region

### Analysis of chloroplast genome hypervariable regions of *Taxodium*

Regions enriched with indels and SNPs can be considered as hypervariable regions in the complete cp genome. Since most indels show periodic variation of repeat motifs, the number of polymorphic sites contained in an indel is affected not only by the degree of periodic variation but also by the size of the repeat motif. When two TRs have the same number of sites, compared with repeats with longer repeat motifs, the repeat sequence with shorter repeat motif has more repeat cycles, indicating a higher polymorphic potential. Therefore, when we select hypervariable regions, these should not only be based on the number of polymorphic sites, but also regions with more indel/SNPs of different origins to ensure that the region has higher polymorphic potential. Based on this principle, the most variable regions of the *Taxodium* cp genome are *clpP*-*accD*, *trnV*-*rps12*, *ndhF*-*trnN*, *trnV*-*ndhC*, *trnI*-*trnL*, *ycf1*-*rpl20*, *atpI*-*atpH*, *rpl36*-*rps11*, *ycf1*, *psbJ*-*clpP*, and *trnI*-*rrn16*(Fig. 2, HR01-HR11). These hypervariable regions contained at least four SNPs and/or indels of diverse origin in this study (Additional file 8). These regions can be considered interspecies mutational hotspots in *Taxodium* and could be potentially high-resolution DNA barcodes in the study of population genetics.

## Discussion

### Phylogenetic and rearrangements analysis

Godfrey [15] considered *T. ascendens* (pond cypress) as a varied form of bald cypress due to temporal differences in phylogeny. Farjon [16] considered that *Taxodium* has two species, *T. distichum* and *T. mucronatum*, but *T. distichum* has two varieties, var. *distichum* and var. *imbricatum* [17]. Both of these views support a closer relationship between baldcypress and pondcypress. However, The phylogenetic trees constructed by chloroplast whole genome sequence and *ycf1* gene sequence here all supported a closer genetic relationship between *T. distichum* and *T. mucronatum*. The closer genetic relationship between *Taxodium* and *Glyptostrobus* is consistent with their similar growth habits. The two genera are both pioneer waterlogging-tolerant tree species, and can develop unique cypress knees acting as pneumatophores thought to help in oxygenation to the roots [11].

SIR> 200 bp are thought to be effective substrates for HR [18]. However, there is a distinct difference between Pinaceae and cupressophyte species, in that the former has more of these sIRs than the latter, and species of the subclade Cupressaceae all have relatively shorter sIRs compared to other two subclades within the cupressophyte clade [11]. Unanimously, cp genome rearrangements are much more frequent in cupressophytes, especially in the subclade Cupressaceae, than in Pinaceae [11]. In our research, only one short sIR greater than 200 bp (282 bp) was found in *T. ascendens*, and we identified at least 13 rearrangements in the process of transformation from *Cycad* to *T. ascendens*, which was consistent with the features of Cupressaceae described above. Suggested mechanisms responsible for cp rearrangements are diverse [8]. Among the 13 inversion endpoints, five located near tRNAs; the same was found in *Trachelium caeruleum* [19]; thus, the rearrangement breakpoints may selectively constrain some regions of the cp genome [20, 21]. Besides, three rearrangement breakpoints were in TRs, suggesting potential association between the rearrangement and repeats.

### Chloroplast genome isomers

The *trnQ*-containing sIRs of Cupressaceae and Taxaceae have been widely proved to be capable of mediating HR, such as the 544 bp sIRs in *Cephalotaxus oliveri* [6], the ~ 250 bp sIR in *Juniperus* [9], and the 298 bp sIRs in *Torreya fargesii* [10]. Although, the *trnQ*-UUG is duplicated, this gene is not retained from the IR region. It is proposed that the *trnQ*-IR originated in the common ancestor of *Cupressaceae* and *Taxaceae* after they split from *Sciadopitysis* and was transformed from the *trnQ*-UUG forward TRs to reverse repeats located in different regions through cp genome rearrangement [7, 12]. Not surprisingly, there is also a 283 bp *trnQ*-containing sIR in *Taxodium* that can mediate HR. There is also a 111 bp *trnI*-CAU containing sIR in *Taxodium*, which was also detected in *Torreya fargesii* [10], Cephalotaxaceae [6], and *Sciadopitys verticillata* [7], but none of them were detected to mediate HR. The *TrnI*-CAU containing sIR was thought to be retained from the ancient IR region [22].

Since, the *trnQ*-IR can mediate HR in *Cupressaceae* and *Taxaceae*, sequences around *trnQ* showed two types of arrangement [9]. In type I, the gene orders were *chlB- trnQ* (UUG)- *trnT* (UGU) & *psbK*- *trnQ* (UUG)-*trnL* (UAA). In type II, gene orders were *chlB- trnQ* (UUG)-*psbK* & *trnT* (UGU) *- trnQ* (UUG)-*trnL* (UAA). Due to the HR mediating by *trnQ*-IR, the two isomers co-exist in individual plant, but the proportions are very different [6, 9, 10]. In some conifers, type I isomer was predominant, for example, *J. scopulorum* has 95% of type I isomer [9]. However, in *Juniperus virginiana*, *Cephalotaxus oliver* and *Ce. wilsoniana* type II isomer was predominant. Just like *J. scopulorum* and *Cryptomeria japonica*, type I isomer is predominant in *Taxodium*, and accounting for 98.65% in *T. ascendens*. The cause of the different contents between the two isomers in individuals is unclear, and the length of sIR may be one factor [10].

### Mutational dynamics in *Taxodium* cp genomes

The relationship between indels and repeats has been widely concerned by many researchers, but they only found that the locations of indels were highly correlated with the location of repeats [6, 14], and they did not study in detail whether indels appeared in the internal, boundary or external region of repeats. This study analyzed the relative location of indels and repeats in detail and found that 83.53% of indels resulted in/caused by periodic variation of repeat motifs, and two indels were accompanied by point mutations of repeat motifs. There are many hypotheses about the molecular mechanism of TR sequence formation, including the unequal exchange hypothesis, the reorganization hypothesis, and the DNA sliding hypothesis. At present, the more accepted view is the sliding hypothesis; that is, TR mutation is the result of the interaction of DNA slippage and DNA replication repair systems. The hypothesis holds that the repeat sequence is formed by the increase in length of the original repeat motif by copy slip, while the original microsatellite sequence may come from a random point mutation. Local de-chaining and re-pairing sometimes occur between the new chains and template chains in the process of replication. When the new strand and template strand are mismatched occasionally, DNA polymerase synthesis on the mismatched DNA strand will cause the length of the new strand to change. If this mismatch is not correctly repaired by the DNA replication repair system in vivo, the next round of replication can produce double-stranded DNA with mutated sequence length [23]. There were 16.47% of indels in *Taxodium* that did not show differences in repetition period. This finding may be explained by the existence of other non-repeat polymerase-stalling sequence motifs; another possible explanation is that repeat sequences were destroyed by mutation, while the indel remained [24].

We identified 72.46% of SNPs around repeat sequences, including 18 locations within repeat sequences and 32 locations within 100 bp adjacent to the TR regions. McDonald et al. proposed that repeat-sequence-induced recurrent repair is the mechanism inducing SNPs [24]. Repeat sequence is one of the main cause of DNA replication fork arrest [25], as a result, DNA polymerases, including high-fidelity DNA polymerase and error-prone repair polymerases, are widely recruited to restart replication [26–28]. Persistent recruitment of error-prone repair polymerases will increase the chance of DNA replication being restarted by an error-prone polymerase and the DNA surrounding the region being synthesized with a higher rate of error [26, 27, 29], leading to an increased likelihood of mutations. A few SNPs located closely to indels, and indel effect may also be an inducement of mutation, which can be explained by the mutagenic-when-heterozygous hypothesis and will vanish over evolutionary time-scales [24].

Highly significant correlations between cp genome polymorphisms (indels and SNPs) and repeats have been reported in some studies [6, 14], but not in others [30]. In our study, although 639 TRs were found to be widely spread on cp genome sequences of *T. ascendens*, only 83 indels and 69 SNPs of diverse origin were detected, leaving no polymorphic sites around most repeats. Not all repeat sequences are surrounded by indels or SNPs, and this may result from the mutation rate of repeat sequences themselves being affected by many other factors, such as repeat sequence length, chromosome location, flanking sequence characteristics, age, etc. [23, 31–33]. Thus, it can be concluded that repeats play a pivotal role in the generation of indel and SNP mutations, but they do not necessarily lead to polymorphism. And we cannot predict mutational hotspot regions based solely on the distribution of repeat sequences.

### Analysis of chloroplast genome hypervariable regions of *Taxodium*

Eleven hypervariable regions of the *Taxodium* cp genome were identified in this study. Among them, *clpP*- *accD* IGS and *ycf1* CDS were the most unique regions. Containing the two largest TRs, they were both the hypervariable regions and arrangement endpoints of *Taxodium*. *AccD* gene and its adjacent sequences were found to extremely expanded due to the insertion of TRs, and they were the hotspots of mutation and rearrangement of cupressophytes [6, 12]. Here, we also analyzed the *ycf1* gene characteristics throughout cupressophytes, and got similar results. The length of the *ycf1* genes showed an extraordinary expansion, and there were universal insertion of TRs. They also located in potential rearrangement endpoints. Besides, in Cupressaceae, the insertion position of TR on *ycf1* and the arrangement of its surrounding genes were quite different from those of other conifers. Thus, similar to *accD* gene and its surrounding regions, *ycf1* gene may play an important role in the cp genome structural evolution of cupressophytes.

## Conclusion

The cp gnomes of *Taxodium* were characterized by several unusual features, such as the loss of the typical IRA copy, the wide spread of TRs, extensive genomic inversions, the presence of isomeric plastomes, and the big variation of *ycf1* genes among genus. All these characteristics highlight the potentially important role of repeats in the dynamics of cp genome mutation and rearrangement. Moreover, the information of TPs and hypervariable regions would provide reliable molecular resources for future research focusing on the infrageneric taxa identification, phylogenetic resolution, population structure and biodiversity for the genus *Taxodium* and Cupressophytes. The comparative chloroplast genomics of the genus *Taxodium* advances our understanding of the dynamics, complexity, and evolution of cp genomes in Cupressophytes.

## Methods

### DNA extraction and sequencing

Fresh buds were harvested from adult trees of *T. distichum*, *T. mucronatum*, and *T. distichum* planted at the Institute of Botany, Jiangsu Province & Chinese Academy of Sciences, Nanjing, China. Total DNA was isolated using an improved extraction method [34]. After DNA isolation, 1 μg of purified DNA was fragmented and used to construct short-insert libraries (insert size 430 bp) according to the manufacturer’s instructions (Illumina), then sequenced on an Illumina Hiseq 4000 [35].

### Genome assembly and annotation

High-quality reads were mapped to the reference cp genome of *T. distichum* (NC_034941) using Bowtie 2 [36] with default parameters. Three coding gene sequence with the highest coverage was used as a seed sequence for de novo assembly of the chloroplast genome by NOVOPlasty v.2.6.2 [37] with the reference genome as a template. Then CAP3 [38] was useds for contigs merging and de-redundancy. Cp cyclization and initiation site determination is done by manual processing.

Cp genes were annotated using an online Dual Organellar GenoMe Annotator tool [39], using default parameters to predict protein-coding genes, tRNA genes, and rRNA genes. The annotation results were further checked and adjusted manually. A whole chloroplast genome Blast [40] search (E-value ≤1e-5, minimal alignment length percentage ≥ 40%) was performed against five databases: Kyoto Encyclopedia of Genes and Genomes (https://www.kegg.jp/), Clusters of Orthologous Groups (http://clovr.org/docs/clusters-of-orthologous-groups-cogs/), Non-Redundant Protein Database, Swiss-Prot (https://web.expasy.org/docs/swiss-prot_guideline.html) and GO (http://geneontology.org/). The circular chloroplast genome map was drawn using OrganellarGenomeDRAW v1.2 [41].

### Estimate of repeats and plastomic inversions

Phobos-v3.3.12 software [42] was used to detect TRs in the cp genomes. The repeat motif length range was set to 1–100 nt (repeat unit length) and the consistency (perfection) was set to be more than 85%. The repeat length of *ycf1* genes TRs with length ≥ 25 bp were detected in *ycf1* genes, and repeat location structure figure were drawn based on the repeat location information. Dispersed repeat sequences were detected by REPuter [22] software, with a minimum size of 30 bp and a maximum of three nucleotides mismatch between the two repeat copies. The repeat sequences were searched using the following four ways: forward, reverse, completion, palindromic.

We downloaded the whole cp genome sequences of 44 species online and from this study, including three species of Pinaceae (Conifers I), 39 cupressophytes (Conifers II), as well as *Ginkgo biloba* and *Cycas taitungensis* as outgroups The 39 cupressophytes covered Cupressaceae, Taxaceae, Sciadopityaceae and Podocarpaceae. Fifty-one single copy coding genes of the 44 species were used for multiple sequence alignment. Mafft version 7 (https://mafft.cbrc.jp/alignment/software/) was used to carry out multiple sequence alignment, and then fasttree 2.1 (http://www.microbesonline.org/fasttree/)was used to build Maximum-likelihood phylogenetic trees. The gene content of related species was visually detected and compared by Mauve [43] with default settings. According to the genome annotation information of each species, sequences information of two genes located before and after *ycf1* genes respectively were extracted, and gene organization containing the five adjacent genes were drawn.

### Detection of isomers

The primers used by Guo et al. [9] were used to test whether the trnQ-containing sIR could mediate homologous recombination. The primer sequences were as follows: rps4 (5′-CCTGGTAAAGTTTTGABACG-3′), psbK (5′-CAAATGAAAAGCGGCATCG-3′), chlB (5′-GTTCCAATATGAGCAGGACCAG-3′), and trnL-UAA (5′-GTTTCCATACCAAGGCTC-3′). PCR was performed using the following primer combinations (rps4 + chlB, rps4 + trnL-UAA, psbK+chlB, psbK+trnL-UAA). Each reaction was 50 ul in volume and included 100 ng DNA. PCR reaction: 94 °C for 3 min → (94 °C for 15 s, 55 °C for 15 s, 72 °C for 1 min) × 35 cycles →72 °C for 3 min → 4 °C for storage. To quantify the relative frequency of the two isomeric genomic forms, Illumina paired-end reads were mapped to the genome using Bowtie 2 [36] with default parameters. The custom Perl script of Guo et al. [9] was used to count repeat-spanning read pairs, enabling us to quantify the frequency of the repeat in each possible genomic arrangement.

### Comparative analysis of genomic structure

InDel refers to the insertion and deletion of sequences in the genome. Samples and reference sequences were compared using LASTZ [44]. The best comparison results were then selected through the processing of axt_correction, axtSort, and axtBest programs, and InDel results were preliminarily obtained. InDel loci were then compared with the sequencing Reads of the samples 150 bp upstream and downstream of the reference sequence using BWA [(http://bio-bwa.sourceforge.net/) software and SAMtools [45] (http://samtools.sourceforge.net/). After filtering, reliable inDels were obtained.

By using MUMmer [46] software, each sample was compared with the reference sequence globally. Sites with differences between the sample sequence and the reference sequence were identified and preliminarily filtered, and potential SNP sites were detected. The sequence of 100 bp on each side of the reference sequence SNP locus was extracted, and then the extracted sequence was compared with the assembly result using BLAST [40] to verify the SNP locus. If the aligned length was less than 101 bp, the unreliable SNP was removed; if the extracted sequence and the assembly result were aligned several times, the SNP considered to be a duplicate region was also removed, and finally a reliable SNP was obtained.

## Supplementary information

**Additional file 1 **Basic information of the 120 genes in *Taxodium distichum*, *Taxodium mucronatum*, and *Taxodium ascenden* chloroplast genomes.

**Additional file 2 **Dot plot analysis of *Taxodium* chloroplast genomes.(A) *Taxodium ascenden &Taxodium distichum*, (B) *Taxodium ascenden &Taxodium mucronatum.*

**Additional file 3 **Tandem repeats detected in the *Taxodium ascendens* chloroplast genome using Phobos software.

**Additional file 4 **Distribution of 1–9 nucleotide repeat motifs with different numbers in *Taxodium ascendens.*

**Additional file 5 **Dispersed repeats detected in the *Taxodium distichum*, *Taxodium mucronatum*, and *Taxodium ascenden* chloroplast genomes.

**Additional file 6 **Mauve alignment of *T. ascendens*, *T. distichum*, *T. mucronatum*, *Glyptostrobus pensilis*, *Cryptomeria japonica*, and *Cycad taitungensis*. Locally collinear blocks are denoted by different color boxes. Histograms within each block represent the degree of sequence similarity.

**Additional file 7 **Information of indels and SNPs in *Taxodium distichum* and/or *Taxodium mucronatum* chloroplast genomes with *Taxodium ascenden* chloroplast genome as reference.

**Additional file 8 **List of the 8 highly variable regions in *Taxodium* chloroplast genomes.

## Data Availability

Sequence information of the 3 cp genomes is available in the NCBI database under the accession number MN535011- MN535013. The reference cp genome of *T. distichum* and the whole cp genome sequences of 44 species analysed in this study were all downloaded from the NCBI database with their accession numbers listed in Fig. 3 and Fig. 5.Other datasets supporting the conclusions of this article are included within the article and its additional files.

## Abbreviations

CDS: Coding DNA sequence
Cp: Chloroplast
HR: Homologous recombination
IR: Inverted repeat
rRNA: Ribosomal RNA
SIR: small inverted repeat
SNP: Single nucleotide polymorphism
SSR: Simple sequence repeat
TR: Tandem repeat
tRNA: Transfer RNA
Ycf1: Hypothetical chloroplast open reading frame 1
