# Supplementary figures and images for: Comparative chloroplast genomics of the genus Taxodium
Source: BMC Genomics. 2020 Jan 31;21:114. doi: 10.1186/s12864-020-6532-1 (PMC6995153; doi:10.1186/s12864-020-6532-1)

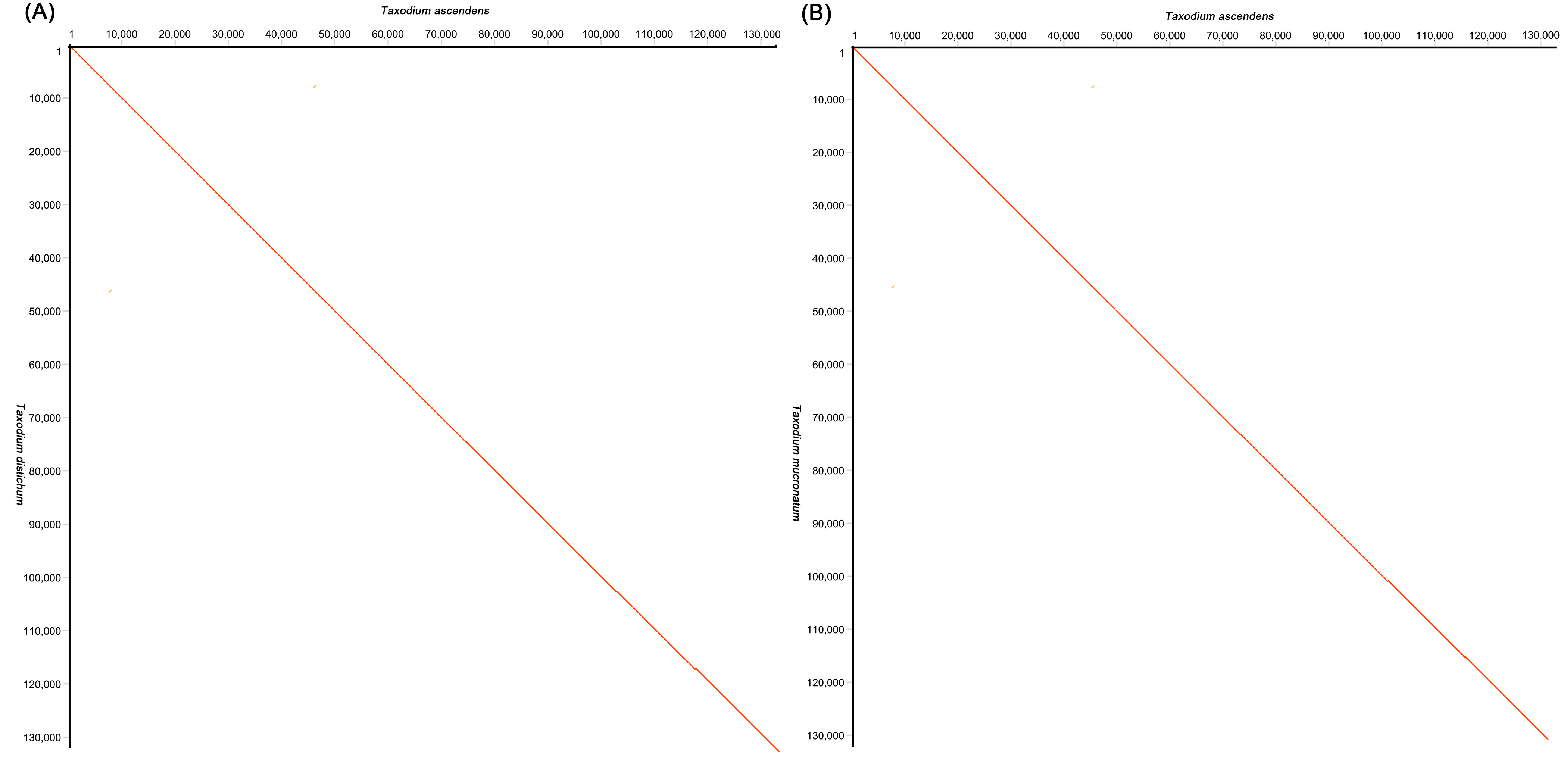

Supplement: Supplementary file 2 — Additional file 2 Dot plot analysis of Taxodium chloroplast genomes.(A) Taxodium ascenden &Taxodium distichum, (B) Taxodium ascenden &Taxodium mucronatum. [file 12864_2020_6532_MOESM2_ESM.jpg]

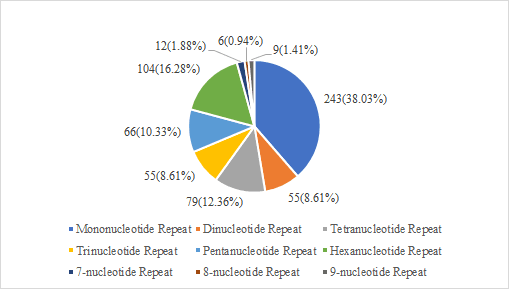

Supplement: Supplementary file 4 — Additional file 4 Distribution of 1–9 nucleotide repeat motifs with different numbers in Taxodium ascendens. [file 12864_2020_6532_MOESM4_ESM.jpg]

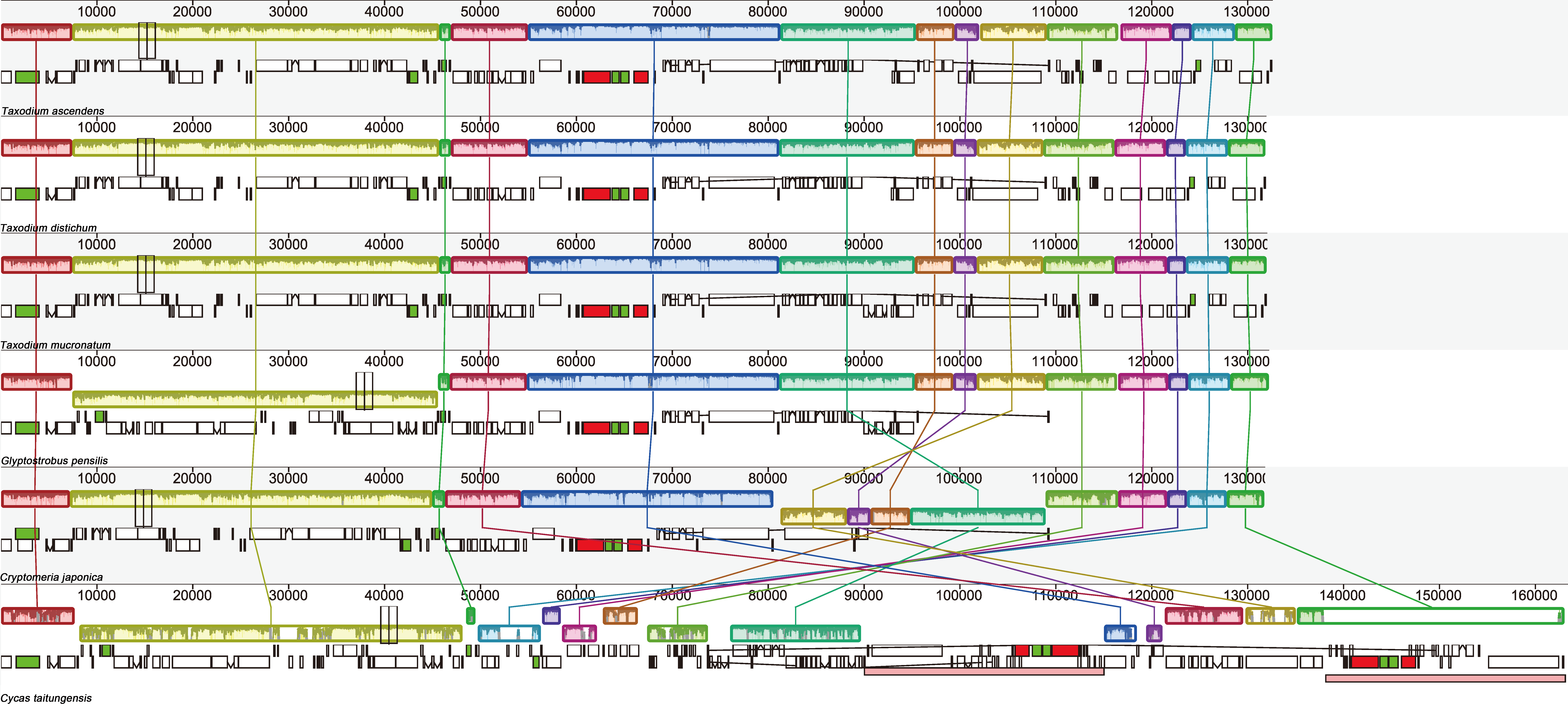

Supplement: Supplementary file 6 — Additional file 6 Mauve alignment of T. ascendens, T. distichum, T. mucronatum, Glyptostrobus pensilis, Cryptomeria japonica, and Cycad taitungensis. Locally collinear blocks are denoted by different color boxes. Histograms within each block represent the degree of sequence similarity. [file 12864_2020_6532_MOESM6_ESM.jpg]
